# Effects of Zearalenone Exposure on the TGF-β1/Smad3 Signaling Pathway and the Expression of Proliferation or Apoptosis Related Genes of Post-Weaning Gilts

**DOI:** 10.3390/toxins10020049

**Published:** 2018-01-23

**Authors:** Min Zhou, Lijie Yang, Minghui Shao, Yuxi Wang, Weiren Yang, Libo Huang, Xuemei Zhou, Shuzhen Jiang, Zaibin Yang

**Affiliations:** 1 Shandong Provincial Key Laboratory of Animal Biotechnology and Disease Control and Prevention, Department of Animal Sciences and Technology, Shandong Agricultural University, 61 Daizong Street, Taian 271018, China; 18854811609@163.com (M.Z.); Yang.superman@163.com (L.Y.); 18853857810@163.com (M.S.); wryang@sdau.edu.cn (W.Y.); huanglibo123@126.com (L.H.); 18206381055@163.com (X.Z.); 2 Agriculture and Agri-Food Canada, Lethbridge Research Centre, Lethbridge, AB T1J 4B1, Canada; yuxi.wang@agr.gc.ca

**Keywords:** zearalenone, proliferating cell nuclear antigen, B-cell lymphoma/leukemia-2, BCL-2 associated X protein, TGF-β1/Smad3 pathway, Our results highlighted an effect of ZEA on uterine proliferation not only through upregulating the expression of PCNA and activating the TGF-β1/Smad3 signaling pathway, but also probably by the accumulated BAX-BCL-2 heterodimers when the level of BCL-2 increased.

## Abstract

Zearalenone (ZEA) is an estrogenic toxin produced by *Fusarium* species, which is widely distributed and posed a great health risk to both humans and farm animals. Reproductive disorders associated with ZEA such as premature puberty, infertility and abortion have plagued the animal husbandry, but the molecular mechanism is unclear. Because transforming growth factor-β1 (TGF-β1) signaling pathway is involved in the proliferation and apoptosis of cells, proliferating cell nuclear antigen (PCNA), B-cell lymphoma/leukemia-2 (BCL-2) and BCL-2 associated X protein (BAX) that all play indispensable roles in the normal development of the uterus, it is hypothesized that ZEA induces reproductive disorders is closely related to the expression of these genes. The objective of this study was to assess the effects of dietary ZEA at the concentrations of 0.5 to 1.5 mg/kg on the mRNA and protein expression of these genes in the uteri of post-weaning gilts and to explore the possible molecular mechanism. Forty healthy post-weaning female piglets (Duroc × Landrace × Large White) aged 38 d were randomly allocated to basal diet supplemented with 0 (Control), 0.5 (ZEA0.5), 1.0 (ZEA1.0), or 1.5 (ZEA1.5) mg/kg purified ZEA, and fed for 35 d. Piglets were euthanized at the end of the experiment and samples were taken and subjected to immunohistochemistry, qRT-PCR and Western blot analyses. The relative mRNA expressions of PCNA, BCL-2 and Smad3 in the uteri of post-weaning gilts increased linearly (*p <* 0.05) and quadratically (*p <* 0.05) as ZEA concentration increased in the diet. The relative protein expressions of PCNA, BAX, BCL-2, TGF-β1, Smad3, and phosphorylated Smad3 (p-Smad3) in the uteri of post-weaning gilts increased linearly (*p <* 0.05) and quadratically (*p <* 0.001) with an increasing level of ZEA. The results showed that uterine cells in the ZEA (0.5–1.5 mg/kg) treatments were in a high proliferation state, indicating that ZEA could accelerate the proliferation of uteri and promote the development of the uteri. At the same time, the results suggested that ZEA activates the TGF-β1/Smad3 signaling pathway, suggesting it plays an important role in accelerating the development of the uterus.

## 1. Introduction

Zearalenone (ZEA), also known as F-2 toxin, is a toxic and low-molecular secondary metabolite mainly produced by *Fusarium* species [1,2,3]. It is widely present in crops and processed products [4,5,6]. The extensive occurrence and high thermal heat stability make ZEA difficult to be eradicated from the food chain, which poses health risks to animals and humans [7,8]. Studies have shown that feeding animals with diets contaminated by ZEA could cause various toxic effects, including the toxicity of reproductive and immune, cytotoxicity, genotoxicity, carcinogenicity, and neurotoxicity [3,9,10,11]. It has been demonstrated that one of the main target organs of ZEA is reproductive system, resulting in atrophy of ovary, atresia of follicle and hypertrophy of uterine wall in female animals [12]. It has been reported that ZEA at the dietary levels of 20 and 40 µg/kg bw in sexually immature gilts induced experimental hyperestrogenism and stimulated the proliferation of nearly all uterine wall tissues [13]. Our previous study also showed that ZEA at the dietary concentrations of 1.1 to 3.2 mg/kg increased genital organ size and hyperplasia of submucosal smooth muscles in the corpus uteri of gilts in a dose-dependent manner [14]. More recently, it has been observed that 1.04 mg/kg ZEA could promote the autocrine action or expression of the ghrelin gene and upregulation of the expression of the proliferating cell nuclear antigen (PCNA) gene in ovary of gilt [15]. However, the mechanism by which dietary ZEA causes reproductive toxicity has not been fully elucidated. Due to the fact that PCNA is usually used as an indicator of cellular proliferation, that B-cell lymphoma/leukemia-2 (BCL-2) gene and BCL-2 associated X protein (BAX) gene both play indispensable roles in regulating apoptosis of cells [16,17,18], and that transforming growth factor-β1 (TGF-β1) signaling pathway [19]. It is hypothesized that ZEA promotes uterine hypertrophy of post-weaning piglets closely related to the expression of PCNA and TGF-β1/Smad3 signaling pathway.

The objective of this study was to explore the molecular mechanism of ZEA induced uterine hypertrophy to provide theoretical basis for further prevention and treatment of reproductive disorders caused by reproductive toxicity of ZEA.

## 2. Results

### 2.1. Relative mRNA Expressions of the PCNA, BAX, BCL-2, TGF-β1, and Smad3 in Uteri of Post-Weaning Gilts

The relative mRNA expressions of PCNA, BAX, BCL-2, TGF-β1 and Smad3 were consistent with those of immunohistochemical analyses (Table 1). The relative mRNA expressions of PCNA, BCL-2 and Smad3 in the uteri of post-weaning gilts increased linearly (*p <* 0.05) and quadratically (*p <* 0.05) as ZEA concentration increased in the diet. The mRNA expressions of PCNA and TGF-β1 for ZEA1.5 and ZEA1.0 treatments were higher (*p <* 0.05) than that of control and ZEA0.5, and that of the ZEA0.5 treatment was also higher (*p <* 0.05) than that of the control. The BAX, BCL-2 and Smad3 expressions were similar among the three ZEA treatments (*p >* 0.05); however, the BAX mRNA expression in the ZEA1.5 treatment, the BCL-2 mRNA expression in the ZEA1.5 and ZEA1.0 treatments, and the Smad3 mRNA expression in the three ZEA treatments were all higher (*p <* 0.05) than that of the control.

### 2.2. Localization of PCNA, BAX, and BCL-2 Immunoreactivity in Uteri of Post-Weaning Gilts

**Figure 1:**
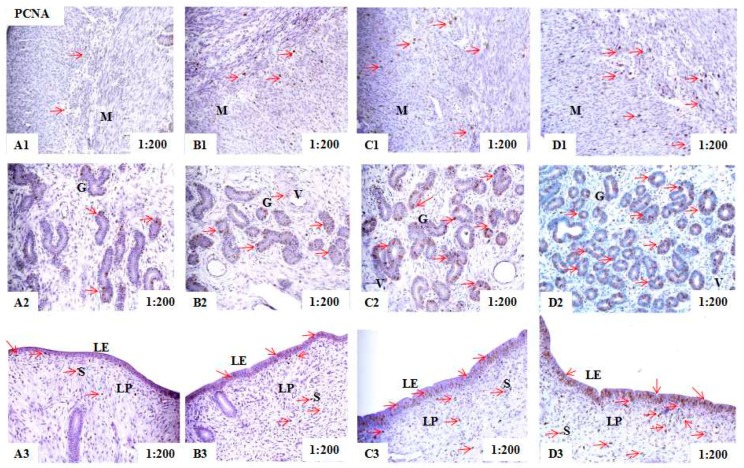
Effects of zearalenone (ZEA) on the proliferating cell nuclear antigen (PCNA) localization in the uteri of post-weaning gilts. Control (**A**), ZEA0.5 (**B**), ZEA1.0 (**C**) and ZEA1.5 (**D**) represent the control diet with an addition of 0, 0.5, 1.0 and 1.5 mg/kg ZEA, and with analyzed ZEA concentrations of 0, 0.52 ± 0.07, 1.04 ± 0.03 and 1.51 ± 0.13 mg/kg, respectively. The 1:200 represents the view of the samples in 200 times. The red arrow represents the immunoreactivity of PCNA. LE was luminal epithelium, G was uterine gland, M was myometrium, S was stromal cells, V was vessel, and LP was lamina propria.

**Figure 2:**
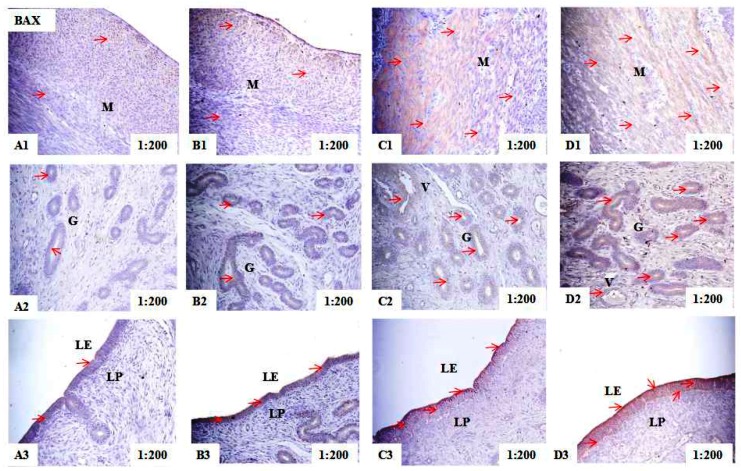
Effects of zearalenone (ZEA) on the BCL-2 (B-cell lymphoma/leukemia-2) associated X protein (BAX) localization in the uteri of post-weaning gilts. Control (**A**), ZEA0.5 (**B**), ZEA1.0 (**C**) and ZEA1.5 (**D**) represent the control diet with an addition of 0, 0.5, 1.0 and 1.5 mg/kg ZEA, and with analyzed ZEA concentrations of 0, 0.52 ± 0.07, 1.04 ± 0.03 and 1.51 ± 0.13 mg/kg, respectively. The 1:200 represents the view of the samples in 200 times. The red arrow represents the immunoreactivity of BAX. LE was luminal epithelium, G was uterine gland, M was myometrium, S was stromal cells, V was vessel, and LP was lamina propria.

**Figure 3:**
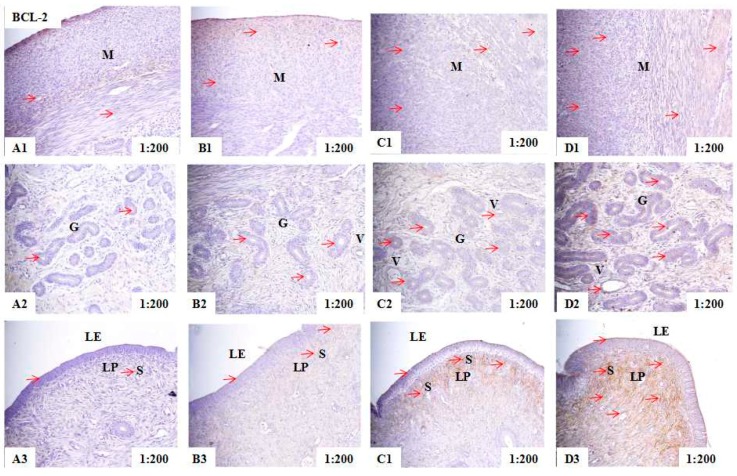
Effects of zearalenone (ZEA) on the B-cell lymphoma/leukemia-2 (BCL-2) localization in the uteri of post-weaning gilts. Control (**A**), ZEA0.5 (**B**), ZEA1.0 (**C**) and ZEA1.5 (**D**) represent the control diet with an addition of 0, 0.5, 1.0 and 1.5 mg/kg ZEA, and with analyzed ZEA concentrations of 0, 0.52 ± 0.07, 1.04 ± 0.03 and 1.51 ± 0.13 mg/kg, respectively. The 1:200 represents the view of the samples in 200 times. The red arrow represents the immunoreactivity of BCL-2. LE was luminal epithelium, G was uterine gland, M was myometrium, S was stromal cells, V was vessel, and LP was lamina propria.

Immunohistochemical analysis showed that PCNA immunoreactive substance was mainly localized in the nuclear of smooth muscle cells (M), glandular epithelial cells (G), luminal epithelial cells (LE), stromal cells (S), and vascular endothelial cells (V) in the uteri of piglets (Figure 1). The BAX immunoreactive substance was mainly localized in the cytoplasm of smooth muscle cells (M), glandular epithelial cells (G), luminal epithelial cells (LE) and vascular endothelial cells (V) in the uteri of piglets (Figure 2). The BCL-2 immunoreactive substance was mainly localized in the cytoplasm of smooth muscle cells (M), glandular epithelial cells (G), luminal epithelial cells (LE), stromal cells (S) and vascular endothelial cells (V) in the uteri of piglets (Figure 3). A light yellow immunoreactive substance of PCNA, BAX and BCL-2 was observed in the control (A). The localization pattern of positive substances in the ZEA-treated pigs was essentially the same as that in the control group (the red arrows). However, compared with the control, the positive reactions of PCNA, BAX, and BCL-2 were enhanced (A1-B1-C1-D1 and A3-B3-C3-D3) (Table 2) in ZEA treated groups, and block localization of yellow and brown immunoreactive substances was observed with an increasing level of ZEA (A2-B2-C2-D2).

The intergrated optic density (IOD) of PCNA, BAX, and BCL-2 in uteri of post-weaning gilts showed linear (*p* < 0.001) and quadratic (*p* < 0.05) increases with an increasing level of ZEA (Table 2). In general, the IOD of PCNA, BAX, and BCL-2 in ZEA1.5 treatment was significantly higher than that of ZEA1.0 treatment (*p* < 0.05), and that of ZEA1.0 treatment was significantly higher than that of ZEA0.5 treatment (*p* < 0.05), and that of ZEA0.5 treatment was significantly higher than that of the control (*p* < 0.05).

### 2.3. Protein Expressions of the PCNA, BAX, BCL-2, TGF-β1, Smad3 and p-Smad3

**Figure 4:**
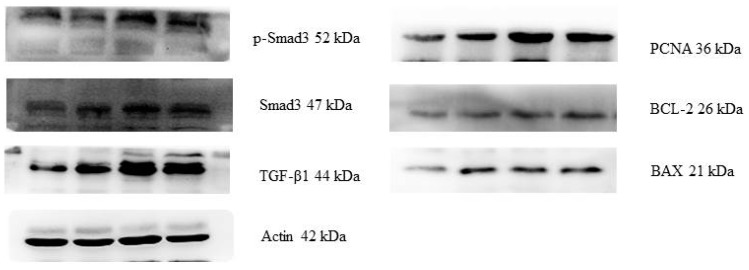
Western blot analysis of proliferation and apoptosis genes, transforming growth factor-β1 (TGF-β1), Smad 3, and p-Smad3 in the uteri of post-weaning gilts.

Western blot analysis revealed the positive bands of appropriate sizes for all of the studied genes (Actin, PCNA, BAX, BCL-2, TGF-β1, Smad3, and p-Smad3) (Figure 4). Results of protein expression were basically consistent with that of the relative mRNA expression (Table 3). The relative protein expressions of PCNA, BAX, BCL-2, TGF-β1, Smad3, and p-Smad3 in the uteri of post-weaning gilts increased linearly (*p <* 0.05) and quadratically (*p <* 0.001) with an increasing level of ZEA. Protein expressions of PCNA, BCL-2 and Smad3 were higher (*p <* 0.05) for ZEA1.5 and ZEA1.0 treatments than for ZEA0.5 treatment and Control groups, which is also true for ZEA0.5 than for Control. However, this difference in protein expressions of PCNA, BCL-2 and Smad3 was not observed between ZEA1.5 and ZEA1.0 treatment groups (*p >* 0.05). The protein expressions of BAX and TGF-β1 in the three ZEA treatments were higher than that of the control (*p <* 0.05). The expression of p-Smad3 was ranked in following order: ZEA1.5 > ZEA1.0 > ZEA0.5 > Control (*p <* 0.05).

## 3. Discussion

The similar growth rate, feed intake, and feed efficiency of the piglets among all the treatments indicated that gilts within a treatment likely consumed a similar amount of digestible energy and other nutrients, and that differences obtained among treatments were likely attributable to the different concentrations of ZEA in the diet [14,20]. 

Success in observation that ZEA promotes the expression of PCNA and activates the TGF-β1/Smad3 signaling pathway in the current study may be very significant. When it comes to the reproductive toxicity of ZEA, it is worth mentioning the effect on the weight and the morphological of uterus. Our previous studies showed that ZEA (0.5–1.5 mg/kg) increased the uterine organ index and increased the thickness of myometrium and endometrium [21], which is basically consistent with previous studies [20,22]. Surprisingly, an immunohistochemical evaluation of apoptosis and proliferation in the mucous membrane of selected uterine in pre-pubertal bitches exposed to low doses of ZEA (50 μg/kg BW and 75 μg/kg BW) provides us a reference [23]. The results of this study showed that apoptotic processes are enhanced only in the epithelial cells lining the uterine endometrium and lower ZEA doses provoke greater proliferative effects than higher doses (e.g., in the lamina propria and uterine glands) [23]. However, slightly different is that our immunohistochemical analysis shows that PCNA immunoreactive substance was mainly localized in the smooth muscle cells, glandular epithelial cells, luminal epithelial cells, and stromal cells in the uteri of piglets and BAX immunoreactive substance was mainly localized in the cytoplasm of smooth muscle cells and luminal epithelial cells. In addition, the IOD of PCNA and BAX in uteri of post-weaning gilts showed linear increases with an increasing level of ZEA. It is worth noting that the mRNA and protein relative expression of PCNA increased linearly as the dietary ZEA concentrations increased from 0.5 to 1.5 mg/kg, which is in accordance with the immunohistochemical. The result indicated that ingested ZEA induced the hyperplasia of endometrium, stroma and perivascular vessels as a result of uterine hypertrophy examined in this study by upregulating the expression of PCNA, so PCNA plays an important role in inducing the development of uteri [24,25]. Research also showed that dietary ZEA at 20 μg/kg and 40 μg/kg levels in sexually immature gilts increased the expression of PCNA and resulted in follicular atresia and apoptosis of granulosa cells [26]. After reaching 120–125 days old, mixed-breed gilts (Large White Polish × Polish Landrance) were fed a diet containing ZEA (200 μg/kg bw and 400 μg/kg bw) for a period of seven days, the cell proliferation of uterus and oviduct were expressed with the growth of PCNA index [27], which was also consistent with the results in the present study. Moreover, previous study reported that ZEA (25 μg/kg bw and 50 μg/kg bw) and its metabolites in bitches caused the degeneration and atropy of granular cell with the result of no reaction for PCNA antigen was observed [28], which also brought great value to this study. Based on the above studies, it was suggested that the effects of ZEA on the expression of PCNA depend on species, age, organ type and dose. Nevertheless, the positive effect of ZEA on uterine hypertrophy of post-weaning piglets further demonstrated alternative molecular mechanisms of ZEA on the estrogen effect.

There are some disputes about the study of ZEA in promoting or inhibiting apoptosis. As we all know, the results were related to many factors. In a series of studies, it has been reported that reduced apoptosis rate of male germ cells and BAX expression as well as an enhancement of BCL-2 expression was a molecular mechanism to against ZEA-induced (10 mg/day) apoptosis in mouse male germ cells [29]. Similarly, it is indicated that a ZEA-mediated anti-apoptotic effect was resultant of the fact that ZEA upregulated the expression of anti-apoptotic gene BCL-2, while downregulated the expression of pro-apoptotic gene BAK in the levels of protein and mRNA [30]. Moreover, a great quantity of studies has shown that the mechanism of inducing apoptosis in some cells was achieved by upregulation of the BAX gene and downregulation of the BCL-2 gene [31,32,33]. Analogously, a study demonstrated that the pregnant sows fed contaminated grain diet (ZEA, 2.77 mg/kg) could induce high expression of BAX and decreased expression of BCL-2 in the uterus [34]. Recently, a high expression of Caspase 9 and 3 was observed in the uterus of female mice exposed to ZEA (0.1 mg/kg) [35], while it has reported that the apoptosis of ovarian granulosa cells caused by ZEA (5 mg/kg) mycotoxicosis in Sprague Dawley (SD) rats is mainly by upregulating the expression of BAX, and the effect of BCL-2 is not obvious [36]. What is worth exploring is that our results are not entirely consistent with the previous studies. What is different from the above studies is that the mRNA and protein relative expression results of BAX and BCL-2 in this study both increased linearly with an increasing level of ZEA. The increasing expression of BAX in this study indicated that ingested ZEA promotes apoptosis of uterine cells to some extent, but the increased BCL-2 neutralized apoptosis via the formation of the heterologous dimer BAX-BCL-2 [37,38]. The above results indicated that the effects of ZEA on reproductive organs were associated with the test method, experimental material, organ type and dose of ZEA. Nevertheless, we proposed that ZEA could promote uterine proliferation not only through upregulating the expression of PCNA, but also probably by the accumulated BAX-BCL-2 heterodimers when the level of BCL-2 increased. However, the mechanism of the above presumption needs to be validated in a following study and we are looking forward to the further test. 

In recent years, the TGF-β signaling pathway has attracted much attention because of its close relationship with inflammation, fibrosis, cancer, proliferation, apoptosis and reproductive activities [19,39]. TGF-β1, the most important factor in the TGF-β family, could regulate endometrial growth, differentiation, embryo implantation and development [40], participate in the apoptosis of endometrial stromal cells together with other cytokines [41], and regulate the transformation of endometrium from proliferation to the secretory phase [42]. The present results show that the mRNA and protein expression of TGF-β1 in the control are significantly lower than that of ZEA treatments, which is accordance with the study that estrogen can increase the expression of TGF-β1 in bone cells [43]. Among the TGF-β1 signals, Smad3 is the key factors that transfer TGF-β1 signal from the extracellular to the nucleus and mediates the biological effects of TGF-β1 at the cellular level [44]. Studies have shown that only phosphorylated Smads (p-Smads) delivered the stimulatory signals of TGF-β1 by directly binding p-Smads with DNA into the nucleus and initiating the transcription of target gene [45]. Unfortunately, within the limits of my ability, no literature on the correlation between ZEA and TGF-β1/Smad3 signaling pathway was observed. It should be noted that the present study revealed, for the first time, the protein expression of TGF-β1, Smad3, and p-Smad3 in ZEA treatments were significantly increased linearly and quadratically with an increasing level of ZEA, suggesting that the TGF-β1/Smad3 signaling pathway was activated and probably involved in the development of uteri. Since other experimental results showed that TGF-β1 could regulate the expression of PCNA in vascular smooth muscle cells [46] and the expression of PCNA and Smad3 in fibroblasts [47]. Furthermore, we put forward a bold hypothesis that the TGF-β1/Smad3 pathway probably provides a feedback that increased the PCNA expression caused by ZEA, and an in vitro test of endometrium epithelial cells treat by ZEA with or without a blocker or activator are in progress. 

## 4. Materials and Methods

### 4.1. Preparation of Zearalenone-Contaminated Diet

Purified crystalline ZEA (Fermentek, Jerusalem, Israel) was dissolved in acetic ether and then poured onto talcum powder. The spiked mixture was left in a fume hood overnight to evaporate acetic ether. The dried mixture contained 1000 mg/kg of ZEA, which was then diluted with toxin-free corn meal to form a premix containing 10 mg/kg of ZEA. The experimental diets were prepared in one batch stored in separate covered containers before feeding. The doses of ZEA used in this study were based on the results of Jiang et al. [20], Chen et al. [14], and Dai et al. [15]. Diets were sampled at the beginning and at the end of the experiment for analyses of mycotoxins and nutrient composition. Deoxynivalenol (DON) was analyzed using high-performance liquid chromatography (HPLC) and an enzyme-linked immunosorbent assay (ELISA). Fluorescent techniques were used to measure ZEA, fumonisins (FUM), and aflatoxin (AFL) levels. The detection limits for AFL, ZEA, DON, and FUM were 1.0 µg/kg, 0.1 mg/kg, 0.1 mg/kg, and 0.25 mg/kg, respectively. 

### 4.2. Animals, Treatments and Feeding Management

The piglets used in all experiments were cared for in accordance with the guidelines for the care and use of laboratory animals prescribed by the Animal Nutrition Research Institute of Shandong Agricultural University and the Ministry of Agriculture of China. A total of 40 healthy post-weaning female piglets (Duroc × Landrace × Large White) at the age of 38 day with average body weight of 14.01 ± 0.86 kg (mean ± SD) were used in this study. Piglets were transferred to individual cages (0.48 m^2^) fitted with plastic slatted floor, feed trough and nipple drinker. The animals were then randomly allocated to one of four dietary treatments, with 10 piglets per treatment. The treatments were basal diet according to National Research Council (NRC) (2012) [48] supplemented with 0 (Control), 0.5 (ZEA0.5), 1.0 (ZEA1.0), or 1.5 (ZEA1.5) mg/kg purified ZEA as described above for 35 d after 10-d adaptation (Table 4). Analyzed ZEA concentrations of the test diets were 0, 0.52 ± 0.07, 1.04 ± 0.03, and 1.51 ± 0.13 mg/kg, respectively. In all treatment diets, no other toxins were detected. Representative samples of feed were taken at the beginning and end of the experimental period for nutrient analyses according to the methods described by the Association of Official Analytical Chemists (AOAC) [49]. Cages were located in an environmental controlled house with the room temperature being set at 30 °C for the first week and thereafter maintained between 26 to 28 °C. The relative humidity was approximately 65% for the entire experimental period. The house was cleaned and disinfected before the start of the experiment.

### 4.3. Sample Collection

At the last day of the feeding trial, piglets were euthanized after being fasted for 12 h. Uteri were immediately isolated from the surrounding fat and tissue under sterile conditions and two samples of uterine tissue from each pig were collected. One sample was collected in an RNase-free 2-mL frozen tube and placed in liquid nitrogen, and then stored at −80 °C for subsequent analysis of the relative mRNA and protein expression of PCNA, BAX, BCL-2, TGF-β1, Smad3 and p-Smad3. Another sample was promptly fixed in Bouin’s solution, followed by sliced into 5-µm sections with a Leica RM 2235 microtome (Leica, Wetzlar, Germany), mounted on poly-l-lysine-coated glass slides, and dried overnight at 37 °C prior to routine staining for immunohistochemical analysis. 

### 4.4. Total RNA Extraction, cDNA Preparation, and Quantitative Real-Time Reverse Transcription Polymerase Chain Reaction (qRT-PCR)

Total RNA was extracted from samples preserved in RNase-free 2-mL frozen tube using RNAiso Plus (Applied TaKaRa, DaLian, China) according to manufacturer’s instructions. The purity and concentration of the RNA was assessed using an Eppendorf Biophotometer (DS-11, Denovix, USA) at an absorbance ratio of 260/280 nm (values in the range 1.8–2.0 indicate a pure RNA sample). The integrity of RNA was verified by agarose gel electrophoresis. Total RNA was reversely transcribed to cDNA using a Reverse Transcription System kit (PrimeScript^TM^ RT Master Mix, RR036A, Applied TaKaRa, DaLian, China).

For qRT-PCR, the total volume of the PCR reaction mixture was 20 µL, which contained SYBR Premix Ex Taq^TM^-TIi RNaseH Plus (TaKara code: RR420A, Lot: AK7502, DaLian, China). Each sample was analyzed in triplicates. The optimized qRT-PCR protocol included an initial denaturation step at 95 °C for 30 s, followed by 43 cycles at 95 °C for 5 s, 60 °C for 34 s, 95 °C for 15 s, 60 °C for 60 s and 95 °C for 15 s. The qRT-PCR reactions were conducted in an ABI 7500 Real Time PCR System (Applied Biosystems, Foster City, CA, USA). The relative amounts of relative mRNA were expressed and calculated as equal to 2^−ΔΔCT^ [50]. The primer sequences and product lengths are presented in Table 5.

### 4.5. Immunohistochemistry

Prior to immunohistochemical analysis, the previously prepared sections were further processed by following procedures. After dewaxing, rehydration and antigen retrieval in sodium citrate buffer (0.01 mol·L^−1^, pH 6.0) using a microwave unit for 20 min at full power, the sections were washed (3 × 5 min) with phosphate buffer saline (PBS) (0.01 mol·L^−1^, pH 7.2). The sections were subsequently incubated in 10% hydrogen peroxide (H_2_O_2_) for 1.5 h to deactivate endogenous peroxidase activity and incubated in 10% normal goat serum (ZSGB-BIO, Beijing, China) for 1 h to block nonspecific binding. 

The immunohistochemical analysis was conducted using commercial kit (Polink-2 plus^®^Polymer HRP Detection system for rabbit or mouse primary antibody, PV-9001/PV-9002, ZSGB-BIO, Beijing, China) with the manufacturer’s recommended procedures. Briefly, the above prepared sections were washed with PBS and incubated with monoclonal mouse antibody PCNA (1:80, ZM-0213, ZSGB-BIO, Beijing, China), monoclonal mouse antibody BAX (1:100, bsm-33279M, BIOSS, Beijing, China) and polyclonal rabbit antibody BCL-2 (1:150, bs-20352R, BIOSS, Beijing, China) at 4 °C overnight. The sections were washed with PBS again and were subsequently incubated in polymer helper at 37 °C for 50 min followed by Polink-2 plus polymer HRP anti-mouse or anti-rabbit at 37 °C for 1 h. After these incubations, the sections were washed with PBS, immersed in diaminobenzidine tetrachloride (DAB kit, TIANGEN PA110, Beijing, China) for 1–3 min, counterstained with hematoxylin and color developed in tap water. The sections were then dehydrated, sealed in clear resin, mounted, and observed microscopically for the distribution of positive cells using a bright field of view.

### 4.6. Measurement of the Integrated Optical Density of the PCNA, BAX, and BCL-2 Immunohistochemistry

Histological sections of the uteri were observed using a microscope (Nikon ELIPSE 80i, Tokyo, Japan) at magnifications of ×200. To evaluate the amount of cell staining and quantity of the target antigen of PCNA, BAX, and BCL-2, the images were analyzed using image analysis software (Image Pro-Plus 6.0, Media Cybernetics, Sliver Spring, MD, USA). This yielded values of the total cross-sectional integrated optical density (IOD) [51], which were used to compare the staining amount in the different treatments. We examined at least five stained sections, which were randomly selected from the 10 piglets in each group.

### 4.7. Western Blotting

The total protein of uterine tissue was extracted by the lysate instructions (Beyotime, Shanghai, China) and detected using a BCA protein assay kit (Beyotime, Shanghai, China) with protein content being adjusted to 50 µg per sample. Samples were separated by electrophoresis on polyacrylamide gels, and were subsequently transferred to immobilon-p transfer membranes (Solarbio, Beijing, China). The membranes were incubated in 10% skimmed milk powder for 2 h, washed with Tris Buffered Saline Tween TBST (pH 7.6) three times, and then added monoclonal anti Actin (1:1000; Beyotime, Shanghai, China), monoclonal mouse antibody PCNA (1:300), monoclonal mouse antibody BAX (1:1000), polyclonal rabbit anti BCL-2 (1:200), rabbit anti-TGF beta 1 (1:300; bs-0086R, BIOSS, Beijing, China), rabbit anti-Smad3 (1:300; bs-3484R, BIOSS, Beijing, China), rabbit anti-Phospho-Smad3 (1:1000; 9520T; Cell Signaling Technology, Boston, MA, USA) primary antibody dilution buffer (Beyotime, Shanghai, China), which was subsequently incubated at 4 °C for overnight. The primary antibody incubated nitrocellulose membrane was washed with TBST, and was then incubated with anti-rabbit IgG (1:3000, Beyotime, Shanghai, China) and anti-mouse IgG antibody (1:4000, Beyotime, Shanghai, China) which were diluted by secondary antibody dilution buffer (Beyotime, Shanghai, China) at 37 °C for 2.5 h, followed by washed with TBST, immersed in a high-sensitivity luminescence reagent (BeyoECL Plus, Beyotime, Shanghai, China), exposed to film using FusionCapt Advance FX7 (Beijing Oriental Science and Technology Development Co. Ltd., Beijing, China), and analyzed using Ipp 6.0 (Image Pro-Plus 6.0, Media Cybernetics, Sliver Spring, MD, USA).

### 4.8. Statistical Analysis

Data of IOD, mRNA and protein expression were subjected to analysis of variance using the general linear model procedure of SAS 9.2 (SAS Institute Inc., Cary, NC, USA). The data were initially analyzed as a completely randomized design with individual piglets as random factors to examine the overall effect of treatments. Orthogonal polynomial contrasts were then used to determine linear and quadratic responses to the ZEA levels of treatments. Significant differences among treatments were further analyzed using Duncan’s multiple range tests. Data were expressed as the mean ± SD. All statements of significance are based on a probability of *p <* 0.05.

## 5. Conclusions

In conclusion, a low dose of ZEA (0.5 mg/kg to 1.5 mg/kg) in the present study could upregulate the mRNA and protein expression of PCNA, BCL-2, TGFβ1, Smad3, and p-Smad3 involved in the regulation of uterine proliferation. Results showed that ZEA (0.5 mg/kg to 1.5 mg/kg) upregulated the expression of PCNA and activated the TGF-β1/Smad3 signaling pathway. It is speculated that the mechanism of ZEA induced uterine proliferation and reproductive disorders may be related to the upregulation of PCNA and the activation of the TGF-β1/Smad3 signaling pathway. Further in vitro studies are needed to determine the relationship between TGFβ1/Smad3 signaling pathway and PCNA in gilts challenged by ZEA.

## Figures and Tables

**Table 1:** Effects of zearalenone on the relative mRNA expressions of PCNA, BAX, BCL-2, TGF-β1 and Smad3 in the uteri of post-weaning piglets.

Items		PCNA	BAX	BCL-2	TGF-β1	Smad3
Control		1.00 ± 0.13 ^c^	1.00 ± 0.11 ^b^	1.00 ± 0.12 ^b^	1.00 ± 0.10 ^c^	1.00 ± 0.15 ^b^
ZEA0.5		1.53 ± 0.19 ^b^	1.23 ± 0.21 ^ab^	1.19 ± 0.08 ^ab^	1.45 ± 0.32 ^b^	1.27 ± 0.08 ^a^
ZEA1.0		2.27 ± 0.14 ^a^	1.37 ± 0.16 ^ab^	1.29 ± 0.21 ^a^	1.99 ± 0.09 ^a^	1.30 ± 0.24 ^a^
ZEA1.5		2.38 ± 0.31 ^a^	1.53 ± 0.42 ^a^	1.41 ± 0.25 ^a^	1.85 ± 0.17 ^a^	1.34 ± 0.07 ^a^
*p*-values	Treatment	<0.001	0.043	<0.001	0.036	0.045
	Linear	<0.001	0.019	<0.001	0.346	0.012
	Quadratic	<0.001	0.067	<0.001	0.649	0.018

Control, ZEA0.5, ZEA1.0 and ZEA1.5 represent the control diet with an addition of 0, 0.5, 1.0 and 1.5 mg/kg ZEA, and with analyzed ZEA concentrations of 0, 0.52 ± 0.07, 1.04 ± 0.03 and 1.51 ± 0.13 mg/kg, respectively; ^a–c^ values with a column with the different letters mean significantly different (*p <* 0.05). PCNA, proliferating cell nuclear antigen. BAX, BCL-2 associated X protein. BCL-2, B-cell lymphoma/leukemia-2. TGF-β1, transforming growth factor-β1. GAPDH, glyceraldehyde-3-phosphate dehydrogenase.

**Table 2:** Effects of zearalenone on the immunoreactive intergrated optic density (IOD) of PCNA, BAX, and BCL-2 in the uteri of post-weaning piglets (×10^3^).

Items		PCNA	BAX	BCL-2
Control		63.18 ± 2.11 ^d^	50.84 ± 2.09 ^d^	51.35 ± 1.10 ^d^
ZEA0.5		71.41 ± 3.16 ^c^	55.19 ± 3.08 ^c^	54.40 ± 2.32 ^c^
ZEA1.0		90.08 ± 5.16 ^b^	59.22 ± 4.21 ^b^	63.19 ± 4.02 ^b^
ZEA1.5		95.53 ± 4.42 ^a^	63.41 ± 3.25 ^a^	68.05 ± 3.17 ^a^
*p*-values	Treatment	0.013	0.032	0.022
	Linear	<0.001	<0.001	<0.001
	Quadratic	<0.001	0.011	0.039

Control, ZEA0.5, ZEA1.0 and ZEA1.5 represent the control diet with an addition of 0, 0.5, 1.0 and 1.5 mg/kg ZEA, and with analyzed ZEA concentrations of 0, 0.52 ± 0.07, 1.04 ± 0.03 and 1.51 ± 0.13 mg/kg, respectively. ^a–d^ values with a column with the different letters mean significantly different (*p <* 0.05). PCNA, proliferating cell nuclear antigen. BAX, BCL-2 associated X protein. BCL-2, B-cell lymphoma/leukemia-2.

**Table 3:** Effects of zearalenone on the relative protein expression of PCNA, BAX, BCL-2, TGF-β1, Smad3 and p-Smad3 in the uteri of post-weaning piglets.

Items		PCNA/Actin	BAX/Actin	BCL-2/Actin	TGF-β1/Actin	Smad3/Actin	p-Smad3/Actin
Control		0.27 ± 0.02 ^c^	0.13 ± 0.02 ^c^	0.11 ± 0.01 ^c^	0.39 ± 0.15 ^b^	0.32 ± 0.01 ^c^	0.24 ± 0.05 ^d^
ZEA0.5		0.62 ± 0.09 ^b^	0.35 ± 0.03 ^b^	0.27 ± 0.02 ^b^	0.91 ± 0.06 ^a^	0.55 ± 0.03 ^b^	0.31 ± 0.08 ^c^
ZEA1.0		0.95 ± 0.10 ^a^	0.43 ± 0.11 ^ab^	0.36 ± 0.01 ^a^	0.99 ± 0.07 ^a^	0.82 ± 0.02 ^a^	0.49 ± 0.06 ^b^
ZEA1.5		1.06 ± 0.08 ^a^	0.46 ± 0.14 ^a^	0.45 ± 0.12 ^a^	0.92 ± 0.05 ^a^	0.75 ± 0.03 ^a^	0.61 ± 0.02 ^a^
*p*-values	Treatment	<0.001	0.002	<0.001	<0.001	<0.001	<0.001
	Linear	<0.001	<0.001	<0.001	0.001	0.014	<0.001
	Quadratic	<0.001	<0.001	<0.001	<0.001	<0.001	<0.001

Control, ZEA0.5, ZEA1.0 and ZEA1.5 represent the control diet with an addition of 0, 0.5, 1.0 and 1.5 mg/kg ZEA, and with analyzed ZEA concentrations of 0, 0.52 ± 0.07, 1.04 ± 0.03 and 1.51 ± 0.13 mg/kg, respectively. ^a–d^ values with a column with the different letters mean significantly different (*p <* 0.05); PCNA, proliferating cell nuclear antigen. BAX, BCL-2 associated X protein. BCL-2, B-cell lymphoma/leukemia-2. TGF-β1, transforming growth factor-β1. p-Smad3, Phosphorylated Smad3.

**Table 4:** Ingredients and nutrient levels of the basal diet (air dry basis) ^(1)^.

Ingredients	Content (%)	Nutrients ^(3)^	
Corn	64.5	Digestible Energy, MJ/kg	13.81
Whey powder	5.0	Crude Protein (%)	19.82
Soybean meal	23.0	Calcium (%)	0.70
Fish meal	5.0	Total Phosphorus (%)	0.64
L-Lysine HCl	0.2	Lysine (%)	1.22
CaHPO_4_	0.7	Sulfur Amino Acid (%)	0.65
Pulverized Limestone	0.3	Threonine (%)	0.75
NaCl	0.3	Trptophan (%)	0.22
Premix ^(2)^	1.0		
Total	100.0		

^(1)^ Treatments were basal diet supplemented with purified ZEA at the level of 0, 0.5, 1.0 or 1.5 mg/kg, with analyzed ZEA concentrations of 0, 0.52 ± 0.07, 1.04 ± 0.03 and 1.51 ± 0.13 mg/kg, respectively; ^(2)^ Supplied per kg of diet: VA 3300 IU, VD_3_ 330 IU, VE 24 IU, VK_3_ 0.75 mg, VB_1_ 1.50 mg, VB_2_ 5.25 mg, VB_12_ 0.026 mg, pantothenic acid 15.00 mg, niacin 22.50 mg, biotin 0.075 mg, folic acid 0.45 mg, Mn 6.00 mg, Fe 150 mg, Zn 150 mg, Cu 9.00 mg, I 0.21 mg, Se 0.45 mg; ^(3)^ Digestible energy was the calculated value, and the other nutrient levels were analyzed value.

**Table 5:** Primer sequences of PCNA, BAX, BCL-2, TGF-β1, Smad3, and GAPDH.

Target Gene	Accession No.	Primer Sequence (5′ to 3′)	Product Size Bp
PCNA	NM_001291925.1	F:GTGATTCCACCACCATGTC R:TGAGACGAGTCCATGCTCG	145
BAX	XM_013998624.2	F:GCCGAAATGTTTGCTGACG R:CAGCCGATCTCGAAGGAAG	156
BCL-2	XM_021077298.1	F:GAGCGTAGACAAGGAGATGC R:TCCGACTGAAGAGCGAAC	239
TGF-β1	AF461808	F:AAAGCGGCAACCAAATCTATGA R:GCTGAGGTAGCGCCAGGAAT	206
Smad3	NM_214137	F:TGGTGCCACGCCACACAGAG R:TCGGGGAGAGGTTTGGAGAA	213
GAPDH	NM_001206359.1	F:ATGGTGAAGGTCGGAGTGAA R:CGTGGGTGGAATCATACTGG	154

PCNA, proliferating cell nuclear antigen. BAX, BCL-2 associated X protein. BCL-2, B-cell lymphoma/leukemia-2. TGF-β1, transforming growth factor-β1. GAPDH, glyceraldehyde-3-phosphate dehydrogenase.
